# The Hidden Burden of Dengue and Chikungunya in Chennai, India

**DOI:** 10.1371/journal.pntd.0003906

**Published:** 2015-07-16

**Authors:** Isabel Rodríguez-Barraquer, Sunil S. Solomon, Periaswamy Kuganantham, Aylur Kailasom Srikrishnan, Canjeevaram K. Vasudevan, Syed H. Iqbal, Pachamuthu Balakrishnan, Suniti Solomon, Shruti H. Mehta, Derek A. T. Cummings

**Affiliations:** 1 Johns Hopkins University, Baltimore, Maryland, United States of America; 2 YRGCARE, Chennai, India; 3 Corporation of Chennai, Chennai, India; Mahidol University, THAILAND

## Abstract

**Background:**

Dengue and chikungunya are rapidly expanding viruses transmitted by mosquitoes of the genus *Aedes*. Few epidemiological studies have examined the extent of transmission of these infections in South India despite an increase in the number of reported cases, and a high suitability for transmission.

**Methods and findings:**

We conducted a household-based seroprevalence survey among 1010 individuals aged 5-40 years living in fifty randomly selected spatial locations in Chennai, Tamil Nadu. Participants were asked to provide a venous blood sample and to complete a brief questionnaire with basic demographic and daily activity information. Previous exposure to dengue and chikungunya was determined using IgG indirect ELISA (Panbio) and IgG ELISA (Novatec), respectively. We used this data to estimate key transmission parameters (force of infection and basic reproductive number) and to explore factors associated with seropositivity. While only 1% of participants reported history of dengue and 20% of chikungunya, we found that 93% (95%CI 89-95%) of participants were seropositive to dengue virus, and 44% (95%CI 37-50%) to chikungunya. Age-specific seroprevalence was consistent with long-tem, endemic circulation of dengue and suggestive of epidemic chikungunya transmission. Seropositivity to dengue and chikungunya were significantly correlated, even after adjusting for individual and household factors. We estimate that 23% of the susceptible population gets infected by dengue each year, corresponding to approximately 228,000 infections. This transmission intensity is significantly higher than that estimated in known hyperendemic settings in Southeast Asia and the Americas.

**Conclusions:**

These results provide unprecedented insight into the very high transmission potential of dengue and chikungunya in Chennai and underscore the need for enhanced surveillance and control methods.

## Author Summary

Despite a recent increase in the number of cases, little data exist on the extent of dengue and chikungunya transmission in Indian cities. We conducted a household-based serosurvey conducted in randomly selected spatial locations across the metropolis of Chennai. We tested samples for evidence of previous infection by dengue and chikungunya viruses and used this data to estimate key transmission parameters (force of infection and basic reproductive number) and to explore factors associated with seropositivity. We found that 93% of participants had been exposed to dengue virus, and 44% to chikungunya. We estimate that 23% of the susceptible population gets infected by dengue virus each year, corresponding to approximately 228,000 infections per year. This transmission intensity is almost three times larger than that in traditionally hyperendemic district in Thailand, and suggests an extremely large proportion of asymptomatic/sub-clinical disease, a lack of recognition of the disease and/or under-reporting.

## Introduction

Dengue and chikungunya are rapidly expanding vector-borne viruses transmitted by mosquitoes of the genus *Aedes*. Little data exist on the extent of transmission in Indian cities via planned seroepidemiological surveys, despite an increase in the reported number of dengue cases, a large Chikungunya outbreak documented in 2005–06 and a high suitability for transmission.[1,2]

Even though dengue has been known to be present in India in for over two centuries, the epidemiology of the disease has changed remarkably over the last two decades, with larger and more frequent outbreaks reported throughout urban and rural areas.[3–5] Co-circulation of the four dengue serotypes was first documented in Delhi in 2003 and, despite a lack of routine virologic surveillance, all serotypes have also been isolated from distinct outbreaks in other regions.[5–7] The predominant presentation of the disease is still non-severe, but several cities have reported increases in the proportion of severe cases. [5,8]. While recent studies have concluded that there is a high probability of dengue occurrence in most of the Indian subcontinent,[1,2] and suggest that only a small fraction of clinically apparent cases are diagnosed and reported[9], there remains uncertainty on the true burden of disease. Few epidemiological studies have measured the extent of transmission at the population level.

Chikungunya was first documented in India in 1963 and outbreaks followed in several Indian states throughout the 1960’s and part of the 1970’s.[10] The disease reappeared in Andhra Pradesh in late 2005 after an apparent absence of over 30 years, and then spread to other states in central and South India throughout 2006.[10]. It is estimated that 1.39 million people developed symptomatic disease during the epidemic but similar to dengue, the true burden of infection is not known.[11]

For diseases like dengue and chikungunya, where the proportion of asymptomatic and mild infections is variable, surveillance based on hospital reports can be misleading and may not reflect the true extent of transmission. Furthermore, differential diagnosis between dengue, chikungunya and other febrile illnesses is often impossible in the absence of adequate serologic and virologic tests, and therefore surveillance data can be subject to important reporting biases in settings where such tests are not common practice. In such instances, seroprevalence studies are needed to adequately quantify and characterize the extent of transmission.

In this paper, we present the results of a household-based serosurvey conducted in 50 randomly selected spatial locations within the south Indian city of Chennai, a city where recent increases in the incidence of dengue and chikungunya have been reported.[3] The objective of this study was to quantify the extent of transmission, by measuring prior exposure of the population to dengue and chikungunya, estimate measures of transmission intensity including the force of infection and the basic reproductive number for each pathogen and to explore factors associated with seropositivity.

## Methods

### Study design

We conducted a household-based serosurvey among individuals living in 50 probabilistically selected spatial locations in Chennai. Serum samples were obtained from individuals aged 5–40 years in selected locations, and tested for IgG against dengue and chikungunya viruses.

### Study setting

Chennai is the capital city of the state of Tamil Nadu in South India. It is located on the east coast of India along the Bay of Bengal and has an average elevation of 6.7 m above sea level. Before the 2011 expansion of the city, Chennai had a population of 4.7 million, a total area of 174 km^2^ and a population density of 26,903 people/km^2^.

Dengue virus and *Aedes aegypti* have been detected regularly in rural and urban Tamil Nadu over the last two decades, with increased transmission usually occurring during monsoon (October–December) and post-monsoon seasons.[3] An outbreak of dengue was documented in Chennai city in 2001 with over 800 reported cases and since then, surveillance efforts have been focused on certain administrative areas considered to be high-risk for dengue.

Chikungunya was absent from Tamil Nadu for over 30 years and re-emerged during the 2005/2006 epidemic.[12] Over 4500 cases were reported, but the real extent of the epidemic remains unknown. Entomologic studies determined that *Aedes aegypti* was the predominant vector during the 2006 epidemic.[11]

### Selection of locations and households

**Fig 1:**
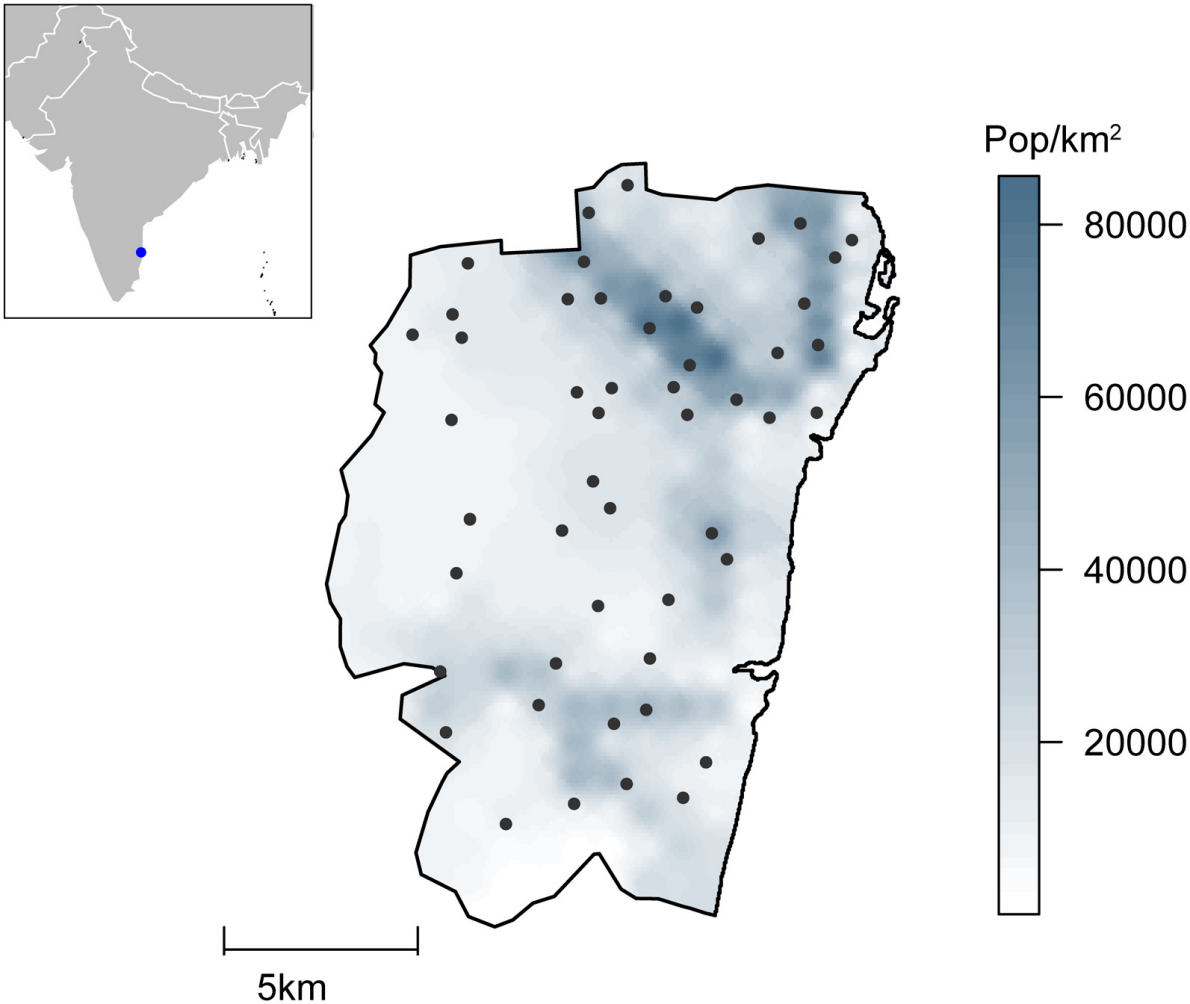
Map of Chennai showing population density estimates and the 50 locations sampled in the study.

A probabilistic sample of fifty locations within Chennai was selected using the LandScan 2010 dataset grid[13], with probability proportional to population count. For each location, random starting coordinates were generated and reviewed using satellite images. Starting points that were located on known non-residential areas (e.g. lakes, parks, train stations), or in which no structures compatible with households were visible within a 200m radius, were rejected and a new starting point was selected from the same location. Fig 1 shows the distribution of selected locations in Chennai city.

Selected locations were visited in random order by the study teams. The household closest to the random starting point was identified using a GPS device and subsequently approached and invited to participate in the study. Recruitment in the same location continued by approaching contiguous households (according to pre-defined rules) until at least 20 participants had been enrolled at each pre-determined location. Due to logistic constrains, enrollment at each location took place during a single day, with the exception of 3 locations where it was not possible to recruit the desired sample size during a single visit. Non-responding households were re-approached up to two times during the day.

### Participants

People living in the selected households were eligible to participate if they were between 5 and 40 years old. Exclusion criteria included medical conditions that contraindicated blood sample collection and inability to give consent. Attempts were made to enroll all eligible participants in each household, even if they were not present at the time of the initial household visit. For example, if the visit took place in the afternoon and the adult male head of the household was away at work, the team would re-visit the household in the evening to collect the missing sample/data. All samples were collected between June and July 2011 by field staff that comprised members from YRGCARE and the Corporation of Chennai.

### Study procedures

Participants were asked to provide a 3ml venous blood sample and to complete a brief questionnaire with basic demographic and daily activity information. They were also questioned about knowledge and past history of dengue and chikungunya. In addition, the head of household was asked to complete a household questionnaire (S2 Text).

Blood samples were collected in anticoagulant-free Vacutainer tubes by trained and certified phlebotomists and transported to the laboratory at YRGCARE where they were centrifuged within 6 hours of collection. Samples were stored at -70±5°C until serological testing.

Historical exposure to dengue and chikungunya and age-specific seroprevalences were determined using Panbio IgG indirect ELISA (Inverness Medical Innovations, Brisbane, Australia) and Novalisa IgG ELISA (Novatec, Germany, product number CHIG0590), respectively. In addition, recent dengue infection was defined using the Panbio IgG Capture ELISA assay. The cut-point of this assay is optimized to detect the high level of IgG antibodies characteristic of acute or recent secondary infections, that are known to last for several months.[14,15] We were only able to test a random subsample of 800 samples for historical dengue exposure due to limited availability of the Panbio IgG indirect Elisa kits. All serological testing was conducted at the YRGCARE laboratory following manufacturer’s instructions. The YRGCARE lab has a NABL (National Accreditation Board for Testing and Calibration Laboratories) certification from the Government of India.

### Statistical analyses

General descriptive statistics were used to explore and compare characteristics of participants and households. To further explore household characteristics we also used a latent-class model.

The force of infection (λ) is the rate at which susceptible individuals are infected and is used to characterize transmission intensity in a given setting. The basic reproductive number (R_0_) is the number of secondary infections generated by a primary infection in a completely susceptible population, and is a measure of transmission potential. To further characterize the extent of transmission of dengue and chikungunya in Chennai, we used the age-specific seroprevalence data to estimate R_0_ for each disease. We also estimated λ and the yearly number of infections for dengue. Details on the methods used to estimate λ, R_0_ and the yearly number of infections are provided in the supplementary material (S1 Text).

We used logistic mixed-effects models to explore the individual and household level factors associated with seropositivity to dengue and chikungunya. All models included a random intercept for location in order to account for potential clustering at these level.

All statistical analyses were performed in R Version 2.14.0.[16]

### Ethical review

This study was approved by the institutional review boards at the YR Gaitonde Centre for AIDS Research and Education (YRGCARE), Chennai and the Johns Hopkins School of Public Health, Baltimore, USA. Written consent was obtained from all adult participants after providing them with a detailed explanation of the study and procedures. Parents/guardians of all child participants were asked to provide written consent on their behalf.

## Results

### Characteristics of participants and households

**Fig 2:**
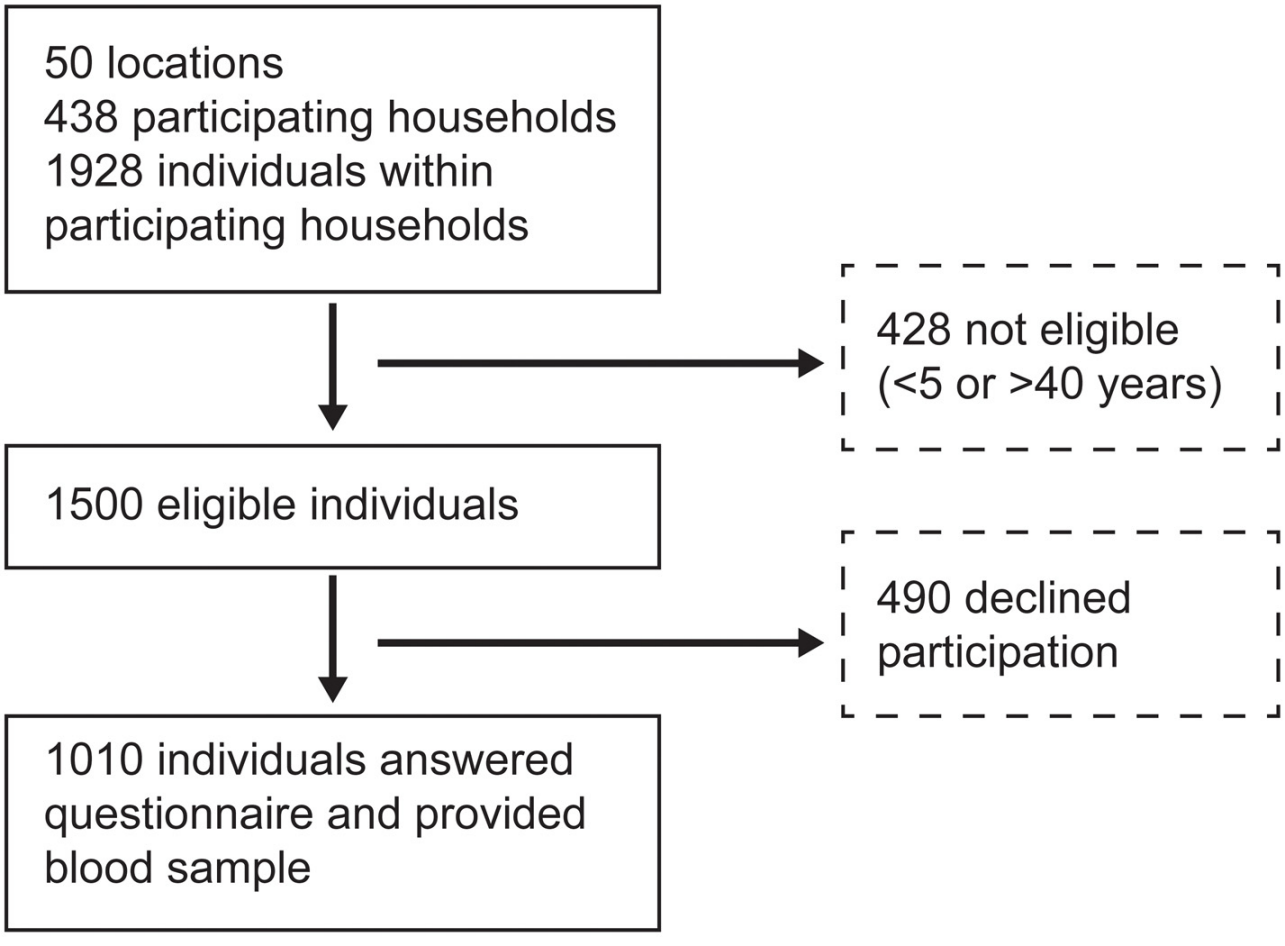
Household and participant enrollment.

Of the 1928 people living in 438 households in the 50 selected locations, 1010 were eligible, consented to participate in the study and provided a blood sample (Fig 2). A median of 9 (Interquartile range [IQR] 8–10) households participated in each location and a median of 2 (IQR 1–3) participants were enrolled per household. The median response rate among participating households was 69% (Range: 33%- 96%). The response rate was lowest in neighborhoods of high socioeconomic stratum and among children aged 5–9 years. In four locations enrollment was stopped before achieving the target sample size due to very low participation rate (<20%).

The median age of participants was 25 years (IQR 15–33 years) and 55% were female (Table 1). There were significant differences in the age distributions across locations but, overall, the age distribution of the enrolled sample was representative of the age distribution of the eligible population in the participating households.

**Table 1:** Characteristics of participants.

	Dengue (Past) (n = 744/800)	Chikungunya (Past) (n = 439/1010)	Overall (n = 1010)
Age *Median (IQR)*	21 (13–30)	25.7 (15.1–34.1)	25 (15–33)
Age Group *n (%)*			
* 5–9*	86 (0.12)	51 (0.12)	132 (0.13)
* 10–14*	101 (0.14)	59 (0.13)	133 (0.13)
* 15–20*	101 (0.14)	59 (0.13)	137 (0.14)
* 20–30*	208 (0.28)	114 (0.26)	272 (0.27)
* 30–40*	248 (0.33)	156 (0.36)	336 (0.33)
Female *n (%)*	112 (0.59)	239 (0.54)	554 (0.55)
Main activity *n (%)*			
* Employee*	190 (0.26)	102 (0.23)	257 (0.25)
* Student*	255 (0.34)	151 (0.34)	349 (0.35)
* Self-employed*	34 (0.05)	22 (0.05)	45 (0.04)
* Unemployed*	21 (0.03)	11 (0.03)	25 (0.02)
* Non-worker*	7 (0.01)	7 (0.02)	18 (0.02)
* Homeworker*	22 (0.03)	21 (0.05)	44 (0.04)
* Housewife*	122 (0.16)	101 (0.23)	224 (0.22)
* Other*	93 (0.13)	14 (0.03)	48 (0.05)
Always lived in same neighborhood	549 (0.74)	353 (0.80)	762 (0.75)
**Awareness of Dengue/Chikungunya**			
Aware of dengue *n (%)*	224 (0.30)	114 (0.26)	290 (0.29)
Aware of chikungunya *n (%)*	432 (0.58)	276 (0.63)	578 (0.57)
History of dengue* *n (%)*	7 (0.01)	6 (0.01)	10 (0.01)
History of Chikungunya* *n (%)*	149 (0.20)	175 (0.40)	202 (0.2)

* Self reported

While all households reported access to electricity and 96% access to an underground drainage system, there was considerable variation in types of dwelling, sources of drinking water, toilet facilities and garbage system (Table 2). Similarly, households came from a broad distribution of monthly incomes. The best fitting latent-class model divided households in four classes (Table A in S1 Text). Additional information about the household characteristics and about the latent-class models is available in the supplementary material.

**Table 2:** Selected characteristics of participating households (n = 438).

Characteristic	N*	%	Characteristic	N	%
Size of household			Main Sources of drinking water^†^		
* 2 or less*	34	0.08	Metro Water		
* 3*	62	0.14	* Piped*	101	0.23
* 4*	175	0.40	* Public Tap*	170	0.39
* 5*	99	0.23	Ground Water		
* 6*	34	0.08	* Hand pump in residence*	51	0.12
* 6 or more*	36	0.08	* Bore Well*	11	0.03
Years living in location			* Pumped into house*	16	0.04
* < = 1*	64	0.15	* Public hand pump*	18	0.04
* 1–5*	107	0.24	* Buy mineral Water*	71	0.16
* 5–10*	65	0.15	* Tanker Truck*	35	0.08
* 10–20*	78	0.18	* From individual*	19	0.04
* >20*	125	0.28	* Water can*	25	0.06
Number of rooms in house			* Other*	6	0.01
* 1*	242	0.55	Toilet		
* 2*	127	0.29	Flush Toilet		
* 3*	52	0.12	* Own*	54	0.12
* 4 or more*	19	0.04	* Shared*	8	0.02
Type of dwelling			Pit toilet/latrine		
* Single House*	160	0.36	* Own*	268	0.61
* Several separate structures*	175	0.40	* Shared*	80	0.18
* Flat/apartment*	94	0.21	* Public*	23	0.05
* Other*	11	0.03	No facility	4	0.01
Monthly household income (Indian Rupees)			Rubbish		
0–2999	26	0.06	* Truck*	289	0.66
3000–4999	94	0.21	* Self-dumping*	117	0.27
5000–6999	98	0.22	* Rubbish pit*	28	0.06
7000–999	76	0.17	* Private collector*	5	0.01
10000–19999	73	0.17			
>20000	70	0.16			
Education (of head of household)					
Professional or honors	8	0.02			
Graduate/Post Graduate	82	0.19			
Intermediate	14	0.03			
Vocational/Trade School	28	0.06			
High school	157	0.36			
Middle School (8th)	74	0.17			
Primary School (5th)	39	0.09			
Not formally educated	8	0.02			
Illiterate	30	0.07			

*Includes data from two additional households that answered questionnaire but did not provide blood samples

^†^Participants were allowed to list up to 2 sources

### Prior exposure to dengue and chikungunya

Overall, 93% of samples (744/800) showed evidence of prior exposure to dengue virus, 44% (439/1010) showed evidence of prior exposure to chikungunya and 41% (325/800) tested positive for both diseases. In addition, high IgG titers were detected in 19% of samples (189/1010) using the capture IgG assay, suggesting recent secondary dengue exposure. Distributions and correlations of the index values obtained using the different testing kits are presented in the supplementary material (Figs A and B in S1 Text). As expected, all samples that tested positive using the capture IgG assay were also tested positive using the indirect assay.

**Fig 3:**
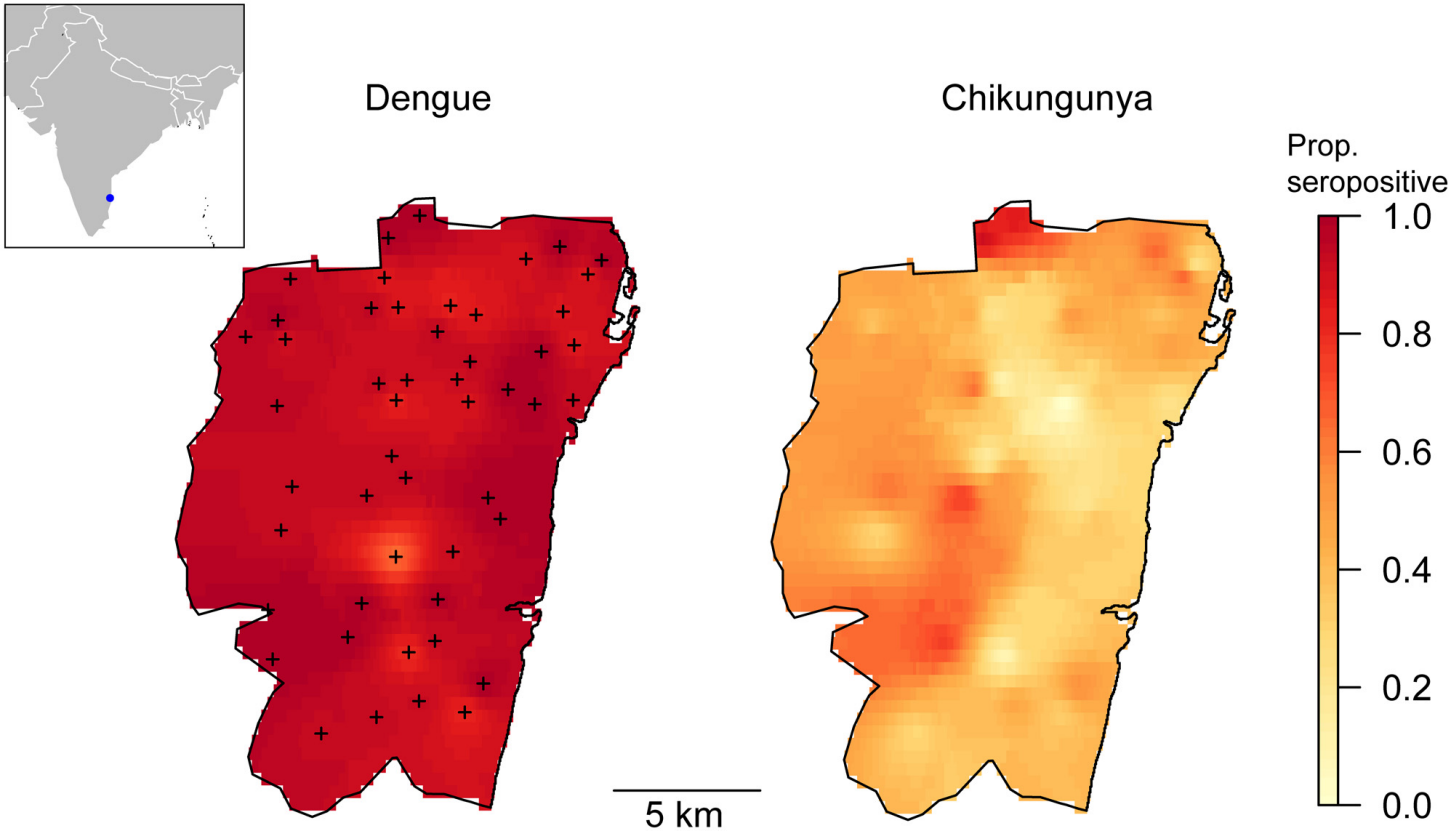
Seroprevalence to dengue and chikungunya by location in Chennai. + symbols indicate sampled locations. Seroprevalence for the rest of locations was interpolated using inverse distance weighting.

While seroprevalence to chikungunya showed great spatial heterogeneity (adjusted ICC = 0.18; 95%CI = 0.11–0.28) with seroprevalences ranging from 0% to 90% in different locations (Fig 3), dengue seropositivity was more spatially homogeneous (adjusted ICC = 0.08; 95%CI = 0.001–0.40) and ranged between 64% and 100%.

**Fig 4:**
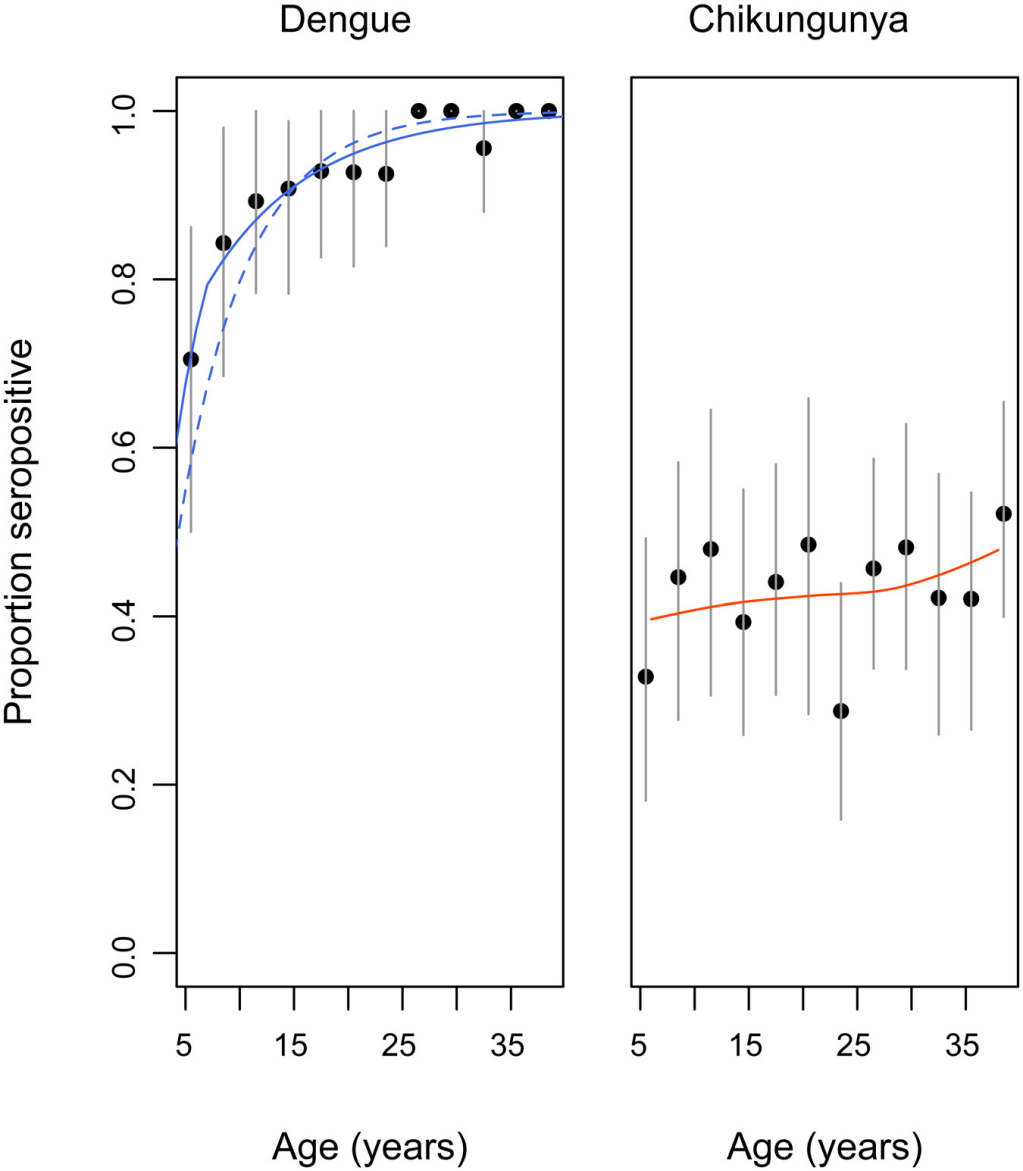
Age-specific seroprevalence to dengue and chikungunya with 95% confidence lines. Data was aggregated into 3-year groups. Dengue: Solid blue lines show the fit of the constant (dashed) and time varying model to the data. Chikungunya: Solid line shows the fit of a Loess smoother.

Fig 4 shows the age-specific seroprevalence curves for both dengue and chikungunya. Dengue seroprevalence increased with age, from 70% (95%CI 50–86%) in children 5–8 years old to 99% (95%CI 97–100%) among adults 25 years of age or older. This pattern suggests endemic circulation of dengue over an extended period of time. In contrast, chikungunya seroprevalence was similar across individuals aged 5–40 years old (42%, 95%CI 3–47%). This pattern suggests that individuals aged 5–40 years in Chennai have experienced a similar cumulative hazard of infection.

Despite the very high seroprevalence of dengue antibodies, only 10 (1%) individuals reported history of dengue. In contrast, 41% of all individuals testing positive for antibodies to chikungunya reported having had chikungunya in the past. All participants who reported having had dengue in the past tested positive for Dengue IgG. As expected, self reported history of chikungunya was also positively associated with being seropositive (p<0.01).

### Force of infection, basic reproductive number and burden of infection

In order to estimate the transmission intensity of dengue, we fit a series of catalytic models to the age-specific seroprevalence data (Table 3), allowing for time constant (Model A) and time varying (Models B and C) forces of infection. The best fitting model was consistent with a significantly higher force of infection during the period 2004–2011 (λ = 0.23; 95%CI 0.16–0.32) preceded by a period of lower, yet positive, transmission (λ = 0.10; 95%CI 0.06–0.15). This result implies that on average, during recent years, 23% of the susceptible population has been infected by dengue each year. In a population as large as Chennai (~4.7 million), we estimate that this hazard leads to approximately 89,700 (95%CI: 77,000–204,200) primary infections and 138,100 (95%CI: 119,700–210,900) secondary infections per year. Fig 4 shows the fit of the time-constant and time varying models to the age-specific seroprevalence data.

**Table 3:** Estimates of the force of infection (λ, summed across all serotypes) and R_0_ obtained from catalytic models fit to dengue age-specific seroprevalence data.

Model*	No. parameters	Estimate		R_0_	p-value
		*Period*	λ		
A	1	*2011-*	0.16 (0.13–0.20)	5.8	-
B	2	*2011–2004*	0.23 (0.16–0.30)	5.8	0.001 ^†^
		*2004-*	0.10 (0.07–0.16)		
C	3	*2011–2003*	0.23 (0.16–0.30)	5.9	0.22 ^γ^
		*2003–1987*	0.07 (0.003–0.15)		
		*1987-*	0.19 (0.04–1.99)		

*We fit models with increasing number of parameters. Thus, while model A assumes that the force of infection has been constant historically, models B and C allow for periods with different transmission intensity.

^†^ p-value of a likelihood ratio test comparing model B vs. model A.

^γ^p-value of a likelihood ratio test comparing model C vs. model B.

**Fig 5:**
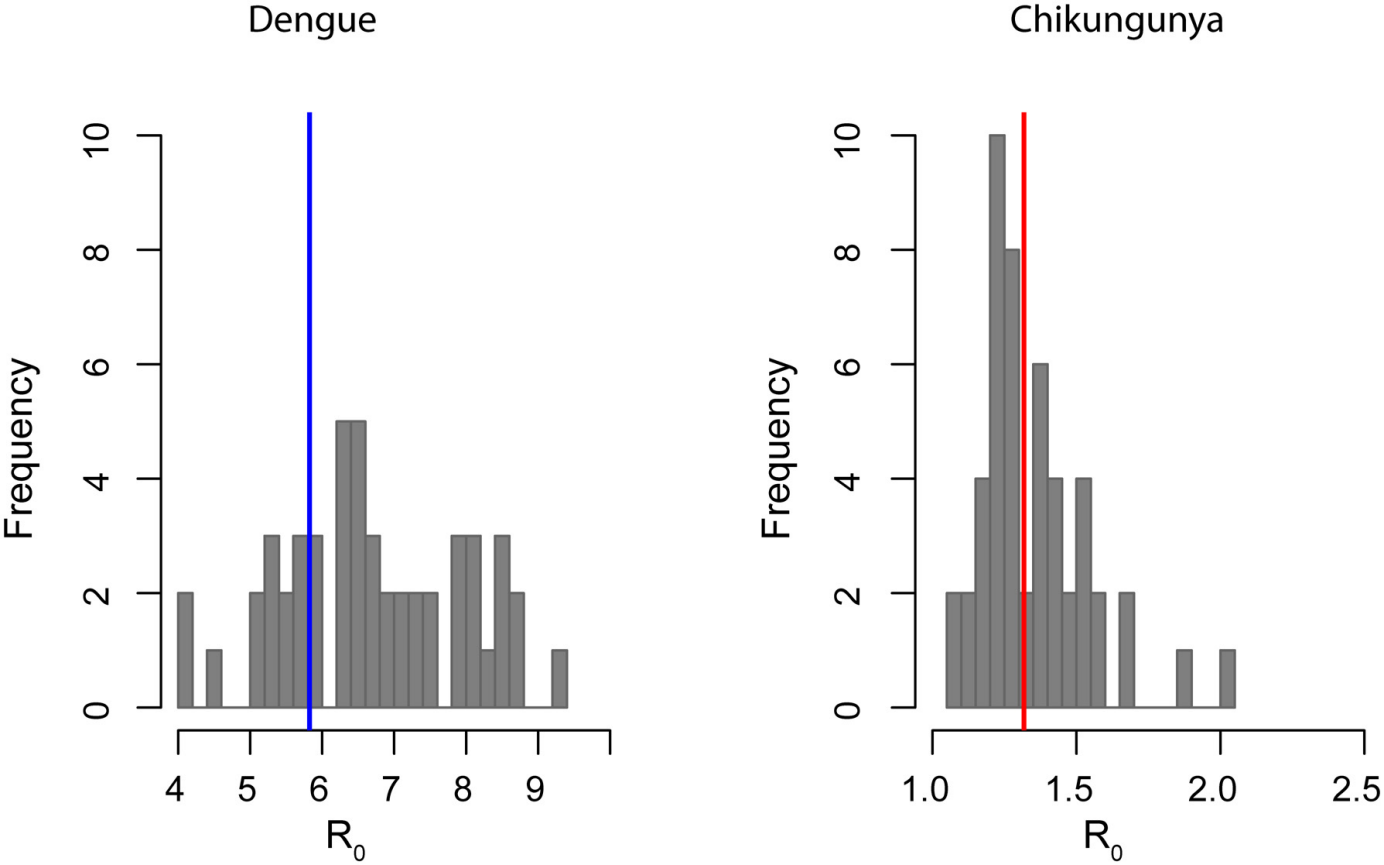
Histogram showing the distribution of location specific R_0_ estimated from the data. Solid lines indicate the average R_0_ estimated for Chennai as a whole.

Fig 5 shows our estimates of the basic reproductive number (R_0_) for dengue in Chennai. These estimates assume endemic circulation of 4 serotypes and were derived using the force of infection estimates and census data. The mean R_0_ for dengue was estimated to be 5.8 per serotype (95%CI 4.9–7.3) but ranged from 4.0 to 9.4 across locations.

Since the uniform age-specific seroprevalence of chikungunya is suggestive of epidemic, rather than endemic circulation, we did not produce estimates of the yearly force of infection. Instead, we estimated R_0_ assuming a single outbreak (probably during 2005/2006). Under this assumption, the mean R_0_ was estimated to be 1.3 (95%CI 1.2–1.40), and ranged between 1.1 and 2.0 between locations.

### Factors associated with prior exposure to dengue and chikungunya

We fit a series of regression models to explore factors associated with seropositivity to dengue and chikungunya (Tables 4 and 5). For each outcome, we selected a final model based on AIC.

**Table 4:** Results of univariate logistic regression of the association between seropositivity to Chikungunya/Dengue and several household and individual factors. All models included a random effect for location.

	Dengue (Past)		Dengue(Recent)		Chikungunya (Past)	
	OR	95% CI	OR	95% CI	OR	95% CI
Age (per year)	3.65	2.47–5.40	0.97	0.96–0.99	1.14	1.01–1.30
Gender-Female	0.75	0.43–1.29	0.80	0.58–1.11	1.09	0.83–1.43
Main activity						
* Employee*	*Ref*	-	*Ref*	-	*Ref*	-
* Student*	0.25	0.11–0.57	1.70	1.12–1.60	2.94	0.62–1.40
Seropositive to chikungunya/dengue	6.22	2.18–10.06			4.92	2.22–10.93
Size of household (per add. member)	1.05	0.88–1.26	1.09	1.00–1.20	1.02	0.94–1.11
Type of dwelling						
* House*	*Ref*	-	*Ref*	-	*Ref*	-
* Separ*. *structures*	1.49	0.74–3.02	1.02	0.71–1.48	1.35	0.96–1.91
* Apartment*	6.75	0.32–1.42	2.85	0.42–19.40	0.50	0.30–0.83
Years lived (per year)	1	0.98–1.02	0.97	0.60–1.57	1.00	0.98–1.02
Source of Drinking Water						
* Metro-Piped*	*Ref*	-	*Ref*	-	*Ref*	-
* Metro-Tap*	1.75	0.81–3.80	1.01	0.64–1.59	1.40	0.91–2.17
* Ground-Hand pump*	4.33	0.92–20.34	1.11	0.60–2.07	1.83	1.05–3.21
* Ground-Bore Well*	-	-	1.51	0.39–5.93	4.02	1.06–1.53
Monthly hh. income (per higher cat)*	0.81	0.67–0.98	0.98	0.88–1.09	0.85	0.77–0.94
Education (Head of household)						
* Graduate*	*Ref*	-	*Ref*	-	*Ref*	-
* High school*	1.01	0.48–2.46	0.87	0.54–1.41	1.91	1.21–3.03
* Illiterate*	0.73	0.23–2.35	0.40	0.16–1.04	1.79	0.91–3.54
Travel in the last month	0.68	0.33–1.41	1.76	1.13–2.75	0.85	0.54–1.32

*Income categories are described in table 2

**Table 5:** Results of multiple logistic regression of the association between seropositivity to Chikungunya/Dengue/Both with household and individual factors. Results of the best fitting model (based on AIC) for each outcome are shown. All models included a random effect for location.

	Dengue (Past)		Dengue (Recent)		Chikungunya (Past)	
	OR	95% CI	OR	95% CI	OR	95% CI
Age (per years)	1.15	1.10–1.20	0.96	0.96–0.99		
Gender-Female			0.73	0.52–1.01		
Years lived (per year)					1.02	1.01–1.04
Seroprevalence (chikungunya/ dengue)	4.26	1.88–9.67	1.46	1.05–2.02	4.44	1.98–9.98
Size of household (per add. member)	1.21	0.99–1.51				
Monthly hh. income (per higher cat)*	0.8	0.63–1.00			0.83	0.74–0.94
Source of Drinking Water						
*Metro-Piped*					*Ref*	-
*Ground-Hand pump*					1.94	1.04–3.66
Travel in the last month			1.87	1.20–2.92		

*Income categories are described in table 2

The main predictor of prior exposure to dengue was age. Dengue seropositivity was also negatively associated with household income (OR 0.8 per higher-income category, 95% CI 0.63, 1.00) but not with any of the other household-level factors. In addition, we found a positive association between reported travel in the last month (by someone in the household) and recent dengue infection (OR 1.94, 95%CI 1.04–3.66). In particular, recent travel within Tamil Nadu or to the adjacent state of Kerala showed statistically significant associations (OR 2.39 (95%CI 1.37, 4.19) and 3.78 (95%CI 1.01, 14.14) respectively.

Chikungunya seropositivity was associated with several household level factors including type of dwelling, years lived in neighborhood, source of drinking water and household income in univariate and adjusted analyses (Tables 4 and 5). Monthly household income was negatively associated with seropositivity (OR 0.83 per higher-income category, 95%CI 0.74–0.94). The odds of seropositivity was 1.94 (95%CI 1.04–3.66) times higher in individuals living in households where the main source of drinking water was a private hand pump (ground water) as compared to those residence who had access to piped water.

Interestingly, an independent predictor of prior exposure to dengue was chikungunya seropositivity and vice-versa, the major predictor of prior exposure to chikungunya was dengue seropositivity. This association was significant even after adjusting for various individual and household-level covariates.

## Discussion

Few epidemiological studies have examined the burden of dengue and chikungunya in South India, despite an increase in the number and frequency of outbreaks of both diseases over the past years. To our knowledge, we present the results of the first household-based serosurvey conducted in the region assessing prior exposure to dengue and chikungunya in the general population.

Our results suggest that 93% of the people aged 5–40 years in Chennai have been exposed to dengue virus. Dengue seropositivity was spatially homogeneous, as expected from the extremely high overall seroprevalence observed, and age-specific seroprevalence was consistent with long-term endemic circulation across the city. We estimate that 23% of the susceptible population gets infected each year, corresponding to approximately 228,000 yearly infections. This hazard is almost three times larger than that estimated recently for a traditionally hyperendemic district in Thailand[17]. Given that only 1% of participants reported having had dengue in the past, these findings are consistent with an extremely large proportion of asymptomatic/sub-clinical disease, a lack of recognition of the disease and/or under-reporting[9].

In contrast, 44% of individuals showed evidence of prior exposure to chikungunya, and seropositivity was spatially heterogeneous. The fact that seropositivity was constant across age groups indicates that individuals 5–40 years of age have been exposed to the same cumulative hazard of infection and is more consistent with epidemic (rather than endemic) transmission. However, since this study did not enroll children younger than 5 years of age and a marker of recent infection is not available, it is not possible to infer whether there has been transmission of chikungunya in Chennai during recent years (2006–2011), following the documented chikungunya reintroduction and outbreak of 2006[18]. Longitudinal studies or new seroprevalence studies including younger age groups will be necessary to ascertain the extent of transmission during recent years.

Seropositivity to dengue and chikungunya were negatively associated with higher household income and, in addition, chikungunya seropositivity was negatively associated with other surrogates of higher socio-economic stratum including access to piped water, living in an apartment and higher educational attainment. Similar associations have been described previously for this and other vector borne diseases.[19–21] These associations with household-level factors along with the observed clustering within locations and households constitute strong evidence in favor of household level transmission of chikungunya and dengue in Chennai.

Given that *Aedes aegypti* has been suggested as the predominant vector for both diseases in Chennai, the observed association between exposure to dengue and chikungunya is not unexpected. Similar associations have been described for these and other infectious diseases that share transmission routes. [22–24] The observed differences in transmission potential of dengue and chikungunya are harder to reconcile. Our estimated R_0_ for dengue, 5.8, is significantly higher than the R_0_s recently estimated by some of our co-authors for hyperendemic settings in Thailand and Brazil [17,25] and comparable only to some estimates of R_0_ in Thailand during the 1980’s and an estimate from Brazil[26–28]. In contrast, estimated R_0_s for chikungunya are lower than previously published estimates from multiple settings.[29,30] Estimates of R_0_ from final epidemic sizes rely strongly on a homogeneous mixing assumption and departures from homogeneity lead to underestimation. Our chikungunya estimates are therefore likely to represent the lower bounds of the true R_0_. If Aedes *aegypti* is truly the principal vector available in Chennai, part of the difference can also be attributed to the known reduced vector competence of this mosquito species for chikungunya transmission [31,32].

A limitation of using IgG ELISA for dengue serological surveys is the known cross-reactivity with other flaviviruses. While Japanese encephalitis (JE) is known to be endemic in several rural districts of India, transmission in Tamil Nadu seems to be limited to several districts in the north and cases diagnosed in Chennai are usually referrals from district level hospitals. It is therefore extremely unlikely that the high dengue IgG seroprevalence measured in this study is attributable to JE. An additional limitation of using IgG based assays is that, in comparison to plaque reduction neutralization assays, they are not serotype specific and do not provide information about the age-specific distribution of monotypic versus multitypic immunity. While in settings of high hyperendemic transmission multitypic immunity accumulates rapidly with age and burden of disease concentrates in children and adolescents, serotype dominance over extended periods of time can lead to pockets of individuals with monotypic immunity in specific age groups. If only a subset of dengue serotypes have circulated in this area (instead of the four serotypes), our estimates of R_0_ are under-estimates of the true R_0_.

Dengue and chikungunya are rapidly spreading arboviral infections in the tropical and sub-tropical settings globally. It is estimated that approximately 2.5 billion people are at risk of dengue alone and that there are 50–100 million infections yearly. [1] Our results are consistent with very high endemic transmission of dengue in Chennai and with more recent, epidemic transmission of chikungunya. These results provide unprecedented insight into the very high transmission potential of dengue and chikungunya in South India and highlight the need for enhanced surveillance and control methods.

## Supporting Information

S1 Text Supplementary methods and results. (PDF)

S2 Text Study questionnaires. (PDF)

S1 Checklist STROBE checklist. (DOC)

S1 Data Dataset containing serological results of the study. (CSV)
