# Supplementary material for: The Hidden Burden of Dengue and Chikungunya in Chennai, India
Source: PLoS Negl Trop Dis. 2015 Jul 16;9(7):e0003906. doi: 10.1371/journal.pntd.0003906 (PMC4504702; doi:10.1371/journal.pntd.0003906)
Supplement: S1 Text — (PDF) [file pntd.0003906.s001.pdf]

Title: The hidden burden of dengue and chikungunya in Chennai, India

Authors: Isabel Rodríguez-Barraquer[1*], Sunil S. Solomon[1,2], Periaswamy Kuganantham[3], Aylur Kailasom Srikrishnan[2], Canjeevaram K. Vasudevan[2], Syed H Iqbal[2], Pachamuthu Balakrishnan[2] , Suniti Solomon[2], Shruti H. Mehta[1], Derek A.T. Cummings[1]

Affiliations: **1.** Johns Hopkins University, Baltimore, Maryland, USA; **2.** YRGCARE, Chennai, India; **3.** Corporation of Chennai, Chennai, India

Supplementary methods and results

Contents

1. Supplementary methods
   1.1. Estimating the force of infection

   1.2. Estimating the basic reproductive number (R_0)

   1.3. Estimating the burden of transmission

2. Supplementary results
   2.1. Latent class analysis

      Table A: Results of latent class analysis. Characteristics of household classes as classified by the latent class model.

   2.2. Figures

      Figure A: Distribution of Index Values obtained with serological assays used.

      Figure B: Correlation between index values obtained with the two dengue assays.

      Figure C: Age-specific seroprevalence to dengue for the two assays used.

1. Supplementary methods

1.1 Estimating the force of infection

The force of infection ($\lambda(t)$) is a measure used to characterize infection hazard in a given setting and estimates the per capita rate of acquisition of infection by susceptible individuals. Assuming that infection is age-independent, age stratified serological surveys provide information about the force of infection over a period of time, as described extensively elsewhere.[1,2]

Since IgG based serological studies do not provide information about infecting serotypes, we estimated forces of infection ($\lambda(t)$) summed across all circulating serotypes.

The proportion of susceptibles aged *a* at time t (x(a,t)) can be expressed as

$$x(a,t) = e^{-\int_0^a \lambda(t-\tau)d\tau}. \qquad (1)$$

Conversely, the proportion of the population that has been exposed (seropositive) can be expressed as 1-x(a,t)

The likelihood of the data is binomial, where the possible outcomes are being susceptible or not.

$$l(\lambda) = \sum_{k=1}^{n}\left[\Delta_x \ln[x(a_k,t_0)] + (1-\Delta_x)\ln\left[1-x(a_k,t_0)\right]\right]. \qquad (2)$$

Here Δ_x represents an indicator variable that takes the value 1 if the person is susceptible (seronegative) and 0 otherwise.

Using the data from the serosurveys conducted in 2011, we first estimated an average, time constant $\lambda(t)$ by maximizing equation 2 using non-linear optimization methods (Model A). We then estimated time varying $\lambda(t)$s by allowing changes in $\lambda(t)$ at pre-specified knots (Models B and C). The optimal location of knots was determined by looking at the profile likelihood assessed at all possible knot-locations. The goodness of fit of models with increasing number of parameters was assessed via a likelihood ratio test of nested models. We approximated 95% confidence boundaries using 500 bootstrap samples.

We then estimated the location specific forces of infection, $\lambda_l(t)$, for the 50 locations sampled. We treated $\lambda_l(t)$, as a random effect, and assumed that λ_l (t), comes from a single underlying distribution.

$$\lambda_l(t) = \lambda_0(t) + u_l$$
$$U \sim N(0, \tau^2)$$

We fit this model using Markov Chain Montecarlo methods using JAGS 3.1.0. We ran three independent chains of 200,000 iterations each (after a burn-in of 50,000 iterations) and checked convergence by visual inspection of the trace plots after achieving a potential scale reduction factor <1.01.

1.2 Estimating the basic reproductive number (R_0)

R_0 is the number of secondary infections generated by a primary case in a completely susceptible population. It is a critical measure of transmission potential because it gives insight into the level of control that is required to reduce incidence and eventually block transmission.

Endemic settings

In settings in which an infectious disease is thought to be in endemic equilibrium, R_0 can be estimated as

$$R_0 = 1 + \frac{B}{A}, \quad (3)$$

where x(t) is the proportion of the population that remains susceptible to the infectious agent. In the case of dengue, where there are up to 4 circulating serotypes, x(t) is replaced by $x_i(t)$, where $x_i(t)$ is the proportion of the population that remains susceptible to serotype *i*. While $x_i(t)$ can't be directly measured, it can be reconstructed using estimates of λ(t). Equation 3 becomes (Ferguson, Donnelly, & Anderson, 1999) :

$$R_{0i} = \frac{1}{x_i(t)} = \frac{1}{\int_0^\infty f(a)[x(a,t) + \sum_{j \neq i} z_i(a,t)]da}, \quad (4)$$

where f(a) is the proportion of the population of age a, ρ represents susceptibility enhancement/inhibition interaction between serotypes, $\sum_{j \neq i} z_1(a,t)$ represents the proportion of people aged a at time t who have been monotypically exposed to other serotypes. This model assumes that people can undergo no more than two infections and that there is no interaction between serotypes.

x(a,t) can be directly reconstructed from equation (1), while $z_i(a,t)$, the probability of being monotypically immune is defined as

$$z_i(a,t) = x(a,t)\left[e^{\int_0^a \lambda / s(t-\tau)d\tau} - 1 \right],(5)$$

where s is the number of circulating serotypes. For all estimates, we assumed endemic circulation of the four serotypes of dengue (s=4). Departures from this assumptions (e.g. circulation of a lower number of serotypes) would lead to even larger estimates of R_0.

Since the serological assay used for this serosurvey does not provide information to estimate serotype specific forces of infection, we were only able to estimate average R_0.

Epidemic settings

For Chikungunya, we produced estimates of R_0 under the assumption that people living in Chennai were exposed to chikungunya during a single, homogeneous epidemic.

This assumption was motivated by the finding that seroprevalence was age-independent, thus suggesting that people aged 5 to 40 years have been exposed to the same cumulative hazard of infection. Under this assumption, R_0 can be estimated from the final epidemic size as follows.

$$R_0 = \frac{-\log(x)}{1-x},(6)$$

where x is the proportion of the population that remains susceptible after the outbreak.

Given that the homogeneous mixing assumption is unlikely to hold, these estimates are likely to underestimate the true R_0 and therefore serve as a lower-bound estimates.
We also produced location specific estimates of R_0 that were based on location specific seroprevalence estimates.

1.3 Estimating the burden of transmission

In an endemic setting, the number of infections expected each year depends on the force of infection and the fraction of the population that remains susceptible.
We estimated the yearly number of dengue infections as,

$$n_{\text{inf}} = \lambda \sum_a x(a)p(a) + \lambda(s-1)\sum_a z_i(a)p(a),(7)$$

where p(a) is the population size of age-class *a.* In this equation, the first term represents the contribution of primary infections and the second term represents the contribution of secondary infections.

References:

1. Grenfell BT, Anderson RM (1985) The estimation of age-related rates of infection from case notifications and serological data. J Hyg (Lond) 95: 419–436.

2. Ferguson NM, Donnelly CA (1999) Transmission dynamics and epidemiology of dengue: insights from age–stratified sero–prevalence surveys. Phil Trans R Soc B. doi:10.1098/rstb.1999.0428.

2. Supplementary results

2. 1 Latent class analysis

To characterize and adjust for household characteristics, we used a latent-class model. We fit multiple models that ranged from 2 to 7 classes. The number of classes in the final model was selected based on the Akaike Information Criterion (AIC). Table A describes the characteristics of the households as classified by the model.

Table A: Results of latent class analysis. Characteristics of household classes as classified by the latent class model. Cells of the table represent proportions unless otherwise specified. Model also included occupancy status and sanitary system.

	**Class 1**	**Class 2**	**Class 3**	**Class 4**
Type of dwelling				
Single House	0.26	0.85	0.15	0.21
Several separate structures	0.64	0.15	0.54	0.21
Flat/apartment	0.0196	0.00	0.3047	0.57
Other	0.08	0.00	0.01	0.01
Number of rooms in house				
1	0.96	0.54	0.53	0.08
2	0.03	0.40	0.46	0.23
3	0.00	0.03	0.00	0.55
4 or more	0.01	0.03	0.01	0.15
Main two sources of drinking water	Public Tap<br>Handpump in residence	Piped into residence<br>Public tap	Piped into residence<br>Public tap	Piped into residence<br>Mineral Water
Toilet				
Flush Toilet	0.0226	0.02	0.04	0.58
Pit toilet/latrine	0.9489	0.98	0.96	0.41
Other	0.0285	0.01	0.00	0.01
Median salary range (Rupees)	3000-4999	5000-6999	7000-9999	>20000
Median educational level (of head of household)	Middle School	Highschool	Highschool	Graduate/ Post-graduate
**Predicted class membership**	**0.19**	**0.27**	**0.27**	**0.19**

2.2 Figures

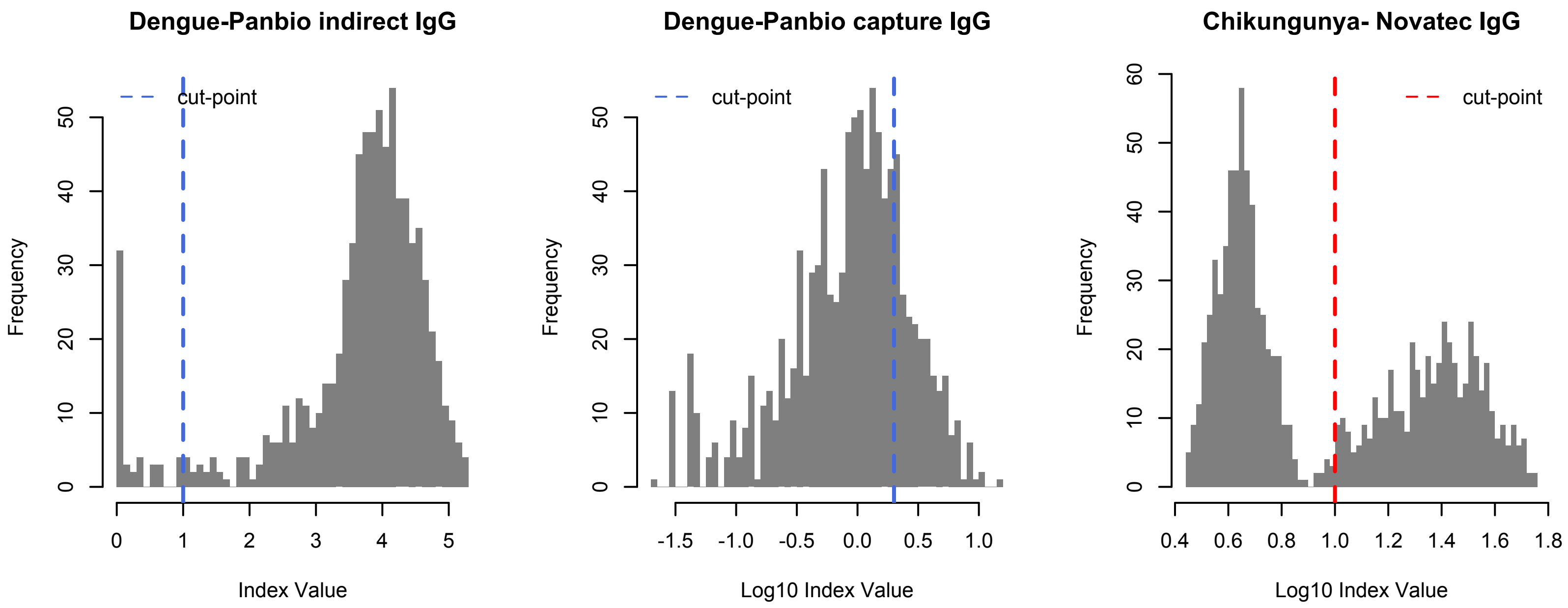

Figure A: Distribution of Index Values obtained with serological assays used. Dashed lines indicate cut-points recommended by the manufacturers.

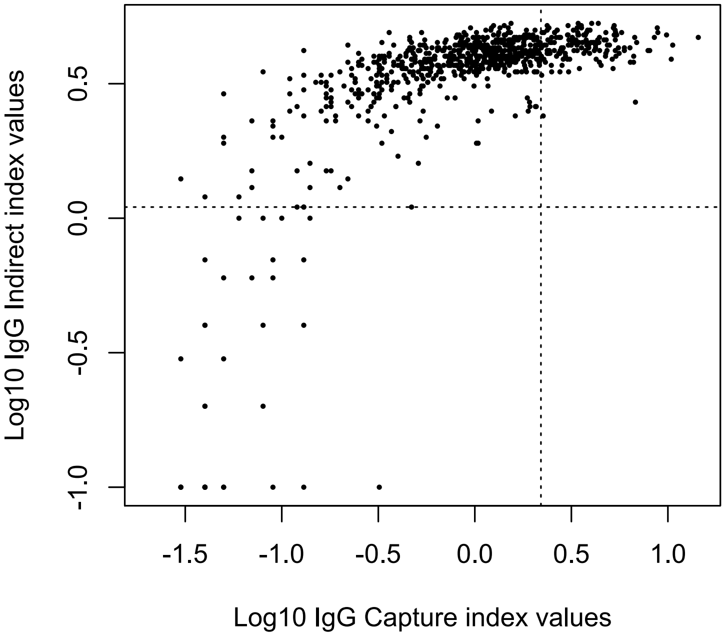

Figure B: Correlation between index values obtained with the two dengue assays. Dotted lines indicate cut-points established by manufacturer.

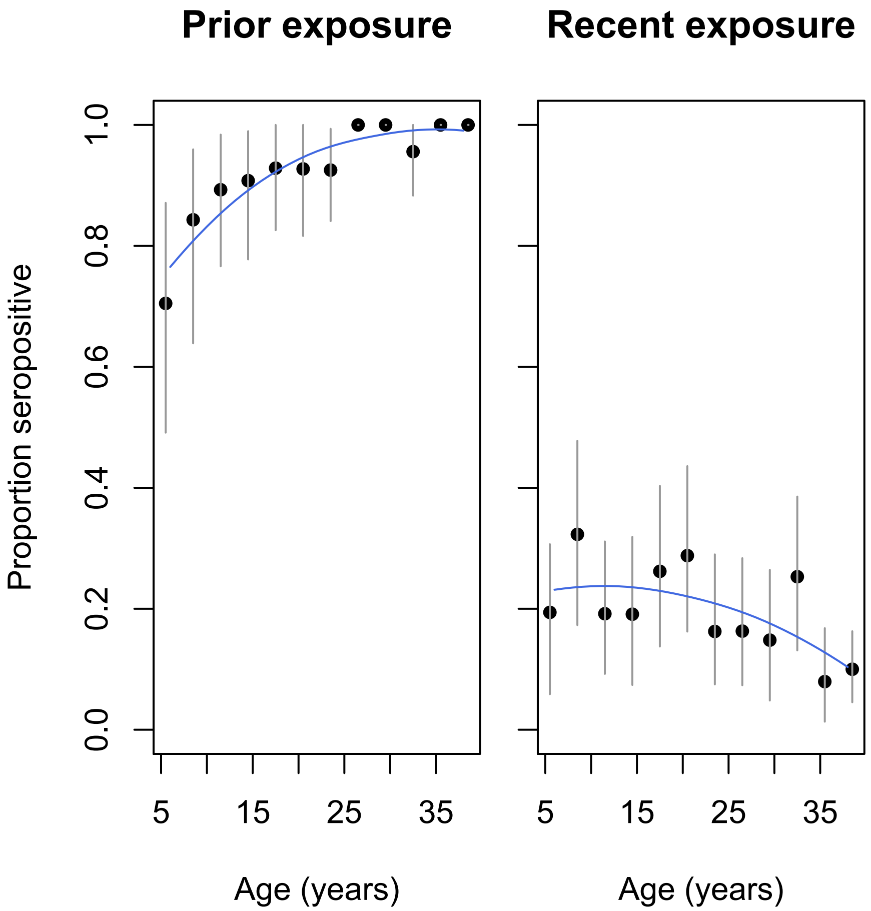

Figure C: Age-specific seroprevalence to dengue for the two assays used. Left: Panbio Indirect IgG (Historical exposure). Right: Panbio Capture IgG (Recent secondary exposure). Lines show the fit of a loess smoother.
