# Supplementary material for: The Hidden Burden of Dengue and Chikungunya in Chennai, India
Source: PLoS Negl Trop Dis. 2015 Jul 16;9(7):e0003906. doi: 10.1371/journal.pntd.0003906 (PMC4504702; doi:10.1371/journal.pntd.0003906)
Supplement: S2 Text — (PDF) [file pntd.0003906.s002.pdf]

The hidden burden of dengue and chikungunya in Chennai, India
Text S2: Questionnaires
Household questionnaire

Dengue/Chikungunya Household information questionnaire.

To be completed by the field researcher during household visit.

Unsure and Decline tick boxes in italic font are there for the field researcher to record if participants give these responses to questions, they should not be read out to participants.

"For the study, we would like to know some general information about your household, such as the number and ages of people living there. Participants can answer these questions together or in private"

Time Started: __________

PARTICIPANTS ARE NOT TO BE ASKED THESE QUESTIONS

1. Household code:

………………………

GPS #: ………………..

Waypoint #: …………….

1.1 Household location:

Longitude: **E**……………………….

Latitude: **N**…………………………

1.2 Zone …………………

A. Members of the household

2. How many members of this household are |___|___| members

A household or dwelling is defined as all living space that shares a kitchen
☐ Unsure ☐ Decline

3. Are there any members of your household who claim this house as their primary residence but are not living here presently?

If no, skip to 6.

4. If yes, where are they presently living?

City/Town: ………………………State: ……………………………… ☐ *Unsure* ☐ *Decline*
City/Town: ………………………State: ……………………………… ☐ *Unsure* ☐ *Decline*
City/Town: ………………………State: ……………………………… ☐ *Unsure* ☐ *Decline*
City/Town: ………………………State: ……………………………… ☐ *Unsure* ☐ *Decline*
City/Town: ………………………State: ……………………………… ☐ *Unsure* ☐ *Decline*

5. Below, for each household member record their age, gender, relationship to each other, and whether they are participant providing social/travel information. Start with the oldest member of the household. If they are a participant, record their participant code too.

	Age/Sex (years)	5-40 years?	Individual questionnaire?	Blood sample?	Participant ID	Comments
Head of Household	………..	☐ Yes<br>☐ No	☐ Yes<br>☐ Declined<br>☐ No	☐ Yes<br>☐ Declined<br>☐ No	……………	
	………..	☐ Yes<br>☐ No	☐ Yes<br>☐ Declined<br>☐ No	☐ Yes<br>☐ Declined<br>☐ No	……………	
	………..	☐ Yes<br>☐ No	☐ Yes<br>☐ Declined<br>☐ No	☐ Yes<br>☐ Declined<br>☐ No	……………	
	……….	☐ Yes<br>☐ No	☐ Yes<br>☐ Declined<br>☐ No	☐ Yes<br>☐ Declined<br>☐ No	……………	
	……..…	☐ Yes<br>☐ No	☐ Yes<br>☐ Declined<br>☐ No	☐ Yes<br>☐ Declined<br>☐ No	……………	
	……..…	☐ Yes<br>☐ No	☐ Yes<br>☐ Declined<br>☐ No	☐ Yes<br>☐ Declined<br>☐ No	……………	
	……..…	☐ Yes<br>☐ No	☐ Yes<br>☐ Declined<br>☐ No	☐ Yes<br>☐ Declined<br>☐ No	……………	
	……..…	☐ Yes<br>☐ No	☐ Yes<br>☐ Declined<br>☐ No	☐ Yes<br>☐ Declined<br>☐ No	……………	
	……..…	☐ Yes<br>☐ No	☐ Yes<br>☐ Declined<br>☐ No	☐ Yes<br>☐ Declined<br>☐ No	……………	
	……..…	☐ Yes<br>☐ No	☐ Yes<br>☐ Declined<br>☐ No	☐ Yes<br>☐ Declined<br>☐ No	……………	
	……..…	☐ Yes<br>☐ No	☐ Yes<br>☐ Declined<br>☐ No	☐ Yes<br>☐ Declined<br>☐ No	……………	
	……..…	☐ Yes<br>☐ No	☐ Yes<br>☐ Declined<br>☐ No	☐ Yes<br>☐ Declined<br>☐ No	……………	

For each participant in the household, please complete an individual form.

Text S2

The hidden burden of dengue and chikungunya in Chennai, India
Text S2: Questionnaires
Household questionnaire

B. Characteristics of the household - I

1 What type of dwelling does the household live in? ☐ *Unsure* ☐ *Decline*
Single house (1)
Several separate structures (2)
Flat/Apartment (3)
Room in larger dwelling (4) |___|
Improvised housing/slum clearance board (5)
Slum (6)
Other (7) ______________________________

2 What is your present occupancy status? ☐ *Unsure* ☐ *Decline*
Owner (1)
Renter (2)
Leasing(3) |___|
Provided free of charge by Relatives/landlord/Employer (4)
Squatting/Living without paying rent (5)

3 How long have you lived at this site? ☐ *Unsure* ☐ *Decline*
If below 12 months, write in the number of months, otherwise write the number of years
_____ months
______ years

4 How many separate rooms do the members of your household occupy? ☐ *Unsure* ☐ *Decline*
(do not count kitchen, bathrooms, toilets, storeroom, pooja rooms, or garage)
_______ rooms

5 Do you have an electricity connection in your house? ☐ *Unsure* ☐ *Decline*
Yes (1)- government approved
Yes (2)- not government approved |___|
No (3)

Text S2

6 What were your **two main** sources of drinking water over the past month?

Metro water
Piped into residence/yard/plot (1)
Public tap (2)

Ground water
Handpump in residence (3)
Bore Well (4)
Pumped into house (5)
Public handpump (6)

Well water
Well in residence/yard/plot
Covered well (7)
Open well (8)
Public well
Covered well (9)
Open well (10)

Surface water
River/stream (11)
Pond/lake (12)

Rainwater (13)
Tanker truck (14)
From individual (15)
Buy mineral water (16)

Other –specify(17)

____

|___| (1)

|___| (2)

☐ *Unsure*
☐ *Decline*

7 Is there a water well within 100meters of the house?

Yes(1) If yes, record longitude and latitude
No (2)- **skip to 8**

|___|

☐ *Unsure*
☐ *Decline*

GPS # _________ Longitude **E**_________________

Waypoint # _______ Latitude **N**_________________

7.1 If yes, is it covered?
Yes-permanently covered (1)
No – not permanently covered (2)

|___|

☐ *Unsure*
☐ *Decline*

Text S2

8 What kind of toilet facility does your household use?

Flush toilet
- Own flush toilet (1)
- Shared flush toilet (2)
- Public flush toilet (3)

Pit toilet/latrine
- own pit toilet/latrine (4)
- Shared pit toilet/latrine (5)
- Public pit toilet/latrine (6)

|___|

No facility/bush/field (7)

Other- specify (8)

____

9 Are you connected to a sanitation system for liquid wastes? ☐ *Unsure* ☐ *Decline*

Yes, underground drains (1)
Yes, open drains (2)
Yes, soak pit/septic tank (3)
Other -specify(4) |___|

____

No (5)

Text S2

10 What kind of rubbish disposal facilities does your household use? ☐ *Unsure* ☐ *Decline*

Collected by truck (1)
Rubbish pit (2)
Burning (3) |___|
Dumped and used for fertilizer (4)
Private collector (5)
Self-dumping (6)
None (7)

11 Name the furthest place any member of the household travelled in the last 6 months ☐ *Unsure* ☐ *Decline*

City/Town: ……………… State: …………………………

If no one has travelled, **skip to 14**

Participant ID: …………………
(if individual is not participant, write relationship with head of household eg. wife, son)

12 Name the furthest place any member of the household travelled in the last 30 days

☐ *Unsure*
☐ *Decline*

City/Town: ……………… State: …………………………

Participant ID: …………………
(if individual is not participant, write relationship with head of household eg. wife, son)

13 Name the furthest place any member of the household travelled in the last 7 days

☐ *Unsure*
☐ *Decline*

City/Town: ……………… State: …………………………

Participant ID: …………………
(if individual is not participant, write relationship with head of household eg. wife, son)

Text S2

14 Do you, or any other member of the household own any of these items (check all that apply)

☐ *Unsure*
☐ *Decline*

☐ Radio
☐ Bicycle
☐ Television
☐ Scooter/bike
☐ Land
☐ Car
☐ Refrigerator
☐ Cellular telephone (working)
☐ Air conditioner

15 What is the estimated total household income per month (averaged over the last year)? Note: all sources of income from any member of the household should be included in this estimate. In Indian rupees |___| ☐ *Unsure* ☐ *Decline*

<1000 (1)
1000-2999 (2)
3000-4999 (3)
5000-6999 (4)
7000-9999 (5)
10000-19999 (6)
>20000 (7)

16 What is your educational level? ☐ *Unsure* ☐ *Decline*

Professional degree or honours (1)
Graduate or Post-graduate (2)
Intermediate (3)
Vocational/Trade School (4)
High school (5)
Middle school(till 8th standard) (6)
Primary school - till 5th standard (7) |___|
Not formally educated (8)
Illiterate (9)
Other -specify(10)

____

17 What is our current occupation? ☐ *Unsure* ☐ *Decline*

Professional (1)
Semi professional (2)
Clerical/shop owner/farm owner (3)
Skilled worker (4)
Semi-skilled worker (5)
Unskilled worker (6) |___|
Unemployed (7)
Other -specify(8)

____

Comments:____
____

Text S2

Text S2

Characteristics of the Dwelling -II

The following questions should me completed by the interviewer.

18 The outer walls of the main dwelling of the household are predominantly made of what material?
Grass (1) |___| ☐ Unsure
Mud (2)
Compacted earth (3)
Mud brick (4)
Burnt bricks (5)
Concrete (6)
Wood (7)
Unfished wood (8)
Iron sheets (9)
Asbestos sheet (10)
Plastic sheets (11)
Cloth (12)
Thatch (palm leafs/ coconut leafs) (13)
Other – specify (14)

____

19 The roof of the main dwelling is predominantly made of what Material? |___| ☐ Unsure
Grass (1)
Iron sheets (2)
Asbestos sheet (3)
Plastic sheets (4)
Cloth (5)
Thatch (palm leafs/ coconut leafs) (6)
Lite roof (7)
Clay tiles (8)
Burnt tiles (9)
Concrete (10)
Plastic (11)
Sheeting (12)
Tarpaulin (13)
Other - specify (14)

____

Text S2

20	The floor of the main dwelling is predominantly made of what material?<br>Sand (1)<br>Smoothed mud (2)<br>Smooth cement (3)<br>Wood (4)<br>Iron sheet (5)<br>Plastic sheet (6)<br>Tile/mosaik (7)<br>Tarpaulin (8)<br>Other – specify (9)<br>____________________________	|___|	☐ Unsure

Text S2

C. Individual questionnaire

Household code……………………….. Participant ID…………………………

Was information on the individual provided by the individual him/herself?
Yes (1) **skip to 2**
No (2)
|___|

1.1 Which household member provided information on behalf of the Individual? (eg. Mother, father, brother)
____
☐ *Unsure*
☐ *Decline*

1.2 Why was the individual not interviewed?
Age under 12 years (1)
Not available to be interviewed at that time (2)
Present, but unwilling to be interviewed (3)
Other –specify (4)
|___|
☐ *Unsure*
☐ *Decline*

____

____

2 Date of birth
|___||___||___|
dd mm yy
☐ *Unsure*
☐ *Decline*

2.1 Age
_________years
☐ *Unsure*
☐ *Decline*

3 Sex
Female (1)
Male (2)
Transgender(3)
|___|
☐ *Unsure*
☐ *Decline*

4 Relationship to head of household
Him/herself (1)
Sibling (2)
Parent (3)
Children (4)
Spouse (5)
Grand –parent (6)
Employee (7)
Other relationship (specify) (8)
|___|
☐ *Unsure*
☐ *Decline*

____

No relationship (9)

Text S2

5	Have you always lived in this neighborhood?<br>Yes (1) –***skip to 6***<br>No (2)		☐ *Unsure*<br>☐ *Decline*
5.1	How long is it since you came to stay here, in this neighborhood/location?<br>*If below 12 months, write in the number of months, otherwise write the number of years*	_______ years<br>_______months	☐ *Unsure*<br>☐ *Decline*
5.2	How long is it since you came to live in this house?<br>*If below 12 months, write in the number of months, otherwise write the number of years*	_______ years<br>_______months	☐ *Unsure*<br>☐ *Decline*
5.3	Where did you move from?<br>Another house, same location/neighborhood (1)<br>Another neighborhood, same zone (2)<br>Another zone, same city (3)<br>Another region/district, same state (4)<br>Another state (5)<br>Another country (6)	|___|	☐ *Unsure*<br>☐ *Decline*
6	What has been your main activity during the last 7 days?<br>Employee (1) - *ask 6.1*<br>Family business worker (2)<br>Self-employed (3)<br>Employer (4)<br>Unemployed, worked before, seeking work (5)<br>Unemployed, worked before, not seeking work (6)<br>Non-worker, never worked before, seeking work (7)<br>Non-worker, never worked before, not seeking work (8)<br>Homeworker (9)<br>Farmer (10)<br>Student (11)<br>Child – not going to school (12)<br>Other (13) -specify<br>________________________________	|___|	☐ *Unsure*<br>☐ *Decline*
6.1	If employee, type of labor<br>Daily wages (1)<br>Weekly wages (2)	|___|	☐ *Unsure*<br>☐ *Decline*

Text S2

Monthly Wages/income (3)
Permanent Income(4)

7	On a typical day, how many hours do you spend away, at a location other than this dwelling?	________ hours	☐ *Unsure* ☐ *Decline*
8	Other than this dwelling, what is the location where you spend most of your day? (name neighborhood) ____________________________		☐ *Unsure* ☐ *Decline*
9	On a typical day (yesterday), do you go to places in your immediate neighborhood, but beyond own house? Yes (1) - time spent (hours) ? No (2)	|___| ___________ hours	☐ *Unsure* ☐ *Decline*
10	On a typical day (yesterday), do you go to places outside your immediate neighborhood but within your zone? Yes (1) - time spent (hours) No (2)	|___| ___________ hours	☐ *Unsure* ☐ *Decline*
11	On a typical day (yesterday), do you go to places outside your zone but within your city? Yes (1) - time spent (hours) No (2)	|___| ___________ hours	☐ *Unsure* ☐ *Decline*
12	On a typical day (yesterday), do you go to places outside your city? Yes (1) - time spent (hours) No (2)	|___| ___________ hours	☐ *Unsure* ☐ *Decline*
13	Are you aware of a disease called dengue? Yes (1) No (2) ***skip to 14***		
	If yes, what do you know? ____________________________ ____________________________	|___|	

Text S2

☐ *Unsure*
13.1 Have you ever had dengue fever in the past? |___| ☐ *Decline*
Yes (1) –date
No (2) ***skip to14*** |___||___||___|
dd mm yy

13.2 If yes, was dengue fever diagnosed at a medical facility? ☐ *Unsure* ☐ *Decline*
Yes (1)
No (2)
|___|

14 Are you aware of a disease called chikungunya?
Yes (1)
No (2)

If yes, what do you know?

____

____

Text S2

14.1 ☐ *Unsure*
Have you ever had Chikungunya in the past? |___| ☐ *Decline*
Yes (1) -date
No (2) - **If no, you have finished the questionnaire** |___||___||___|
dd mm yy

14.2 If yes, was Chikungunya diagnosed at a medical facility? |___| ☐ *Unsure* ☐ *Decline*
Yes (1)
No (2)

Comments:

____

____

____

THANK YOU VERY MUCH !!!!
